# What principles should guide interactions between population health researchers and the food industry? Systematic scoping review of peer‐reviewed and grey literature

**DOI:** 10.1111/obr.12851

**Published:** 2019-04-09

**Authors:** Katherine Cullerton, Jean Adams, Nita Forouhi, Oliver Francis, Martin White

**Affiliations:** ^1^ Centre for Diet and Activity Research, MRC Epidemiology Unit University of Cambridge Cambridge UK; ^2^ School of Public Health University of Queensland Herston Australia

**Keywords:** conflict of interest, food industry, nutrition, public‐private partnerships, research

## Abstract

There is no explicit consensus amongst population health researchers regarding what constitutes acceptable or effective interactions with the food industry. This has led to confusion and disagreements over conflicts of interest, which can undermine the integrity of science. To clarify this issue, we aimed to systematically identify the key principles developed by population health researchers to prevent or minimize conflicts of interest when interacting with the food industry. Databases of peer‐reviewed literature were searched. In addition, an advanced Google search, a request to experts seeking related documents, and hand searching of references were undertaken. Thematic analysis of the extracted data was undertaken. We examined 54 eligible documents describing guidelines for population health researchers when interacting with the food industry. Fifty‐six principles were identified and synthesized in five themes. There were high levels of agreement in themes relating to research governance, transparency, and publication but less agreement and guidance on how principles should be applied in relation to funding and risk assessment. There is agreement on some of the general principles for preventing and minimizing conflicts of interests for population health researchers when interacting with the food industry. However, for issues such as assessing the appropriateness of an industry partner, greater clarity and consensus are required.

## 1 INTRODUCTION

Poor diet is a growing global public health challenge, with important implications for non‐communicable diseases.^1^ Throughout the world, a substantial proportion of the food we eat now comes from the commercial food system.^2^, ^3^ Achieving healthier diets in populations will require action by food industry players, either voluntary or mandated. Such action may have the potential to be strengthened by interactions between the food industry and public sector researchers who work in the field of diet and population health, including those who study nutritional epidemiology, public health nutrition, and dietary behaviours at a population level (who we refer to hereafter as “population health researchers”). These interactions are often actively encouraged by funding bodies and research institutions.^4^, ^5^ However, the primary purposes of the food industry (to maximize profit) and population health researchers (to further knowledge to inform health improvement) are often poorly aligned, leading to the potential for conflicts of interest. These real and perceived conflicts of interest can undermine the credibility of research and researchers, resulting in an erosion of trust amongst the general public and policy makers and scepticism of published research.^6^, ^7^, ^8^, ^9^

Despite these concerns, there is currently no explicit international consensus for population health researchers regarding what constitutes acceptable or effective interactions with the food industry.^10^, ^11^ A significant factor informing acceptable interactions is preventing the damaging impacts of actual or perceived conflict of interests. Whilst guidance regarding acceptable interactions and conflicts of interest involving the food industry has been developed for non‐government organizations^12^ and for policy makers,^13^, ^14^ limited work has been undertaken specifically for population health researchers, addressing the unique challenges they face. Consensus‐driven principles for nutrition researchers (including those working in population health) have been developed in the United States of America by representatives of US government agencies, the food industry, and professional nutrition associations to guide research partnerships.^15^, ^16^, ^17^ However, the involvement of the food industry in the development of researcher engagement principles is considered problematic by many.^8^, ^18^ The absence of clear consensus on what are acceptable interactions between population health researchers and the food industry has given rise to disagreements and confusion, which can further undermine credibility and integrity of nutritional and dietary public health science, further eroding trust and exacerbating scepticism.

Confusion remains for a number of reasons. Firstly, there is a lack of clarity around the term “conflict of interest.” One commonly cited definition of conflict of interest is “a set of conditions in which professional judgment concerning a primary interest (such as a patient's welfare or the validity of research) tends to be unduly influenced by a secondary interest (such as financial gain).”^19^ Financial gain tends to be the focus for conflict of interest guidance. 20 However, financial gain is not the only factor that may lead to conflicts of interest for researchers. The desire for recognition, academic advancement, and success in publication and funding are other powerful influences.^21^ Furthermore, conflicts of interest can also arise from researcher's personal relationships and business associations.^11^ A conflict of interest can also be perceived to exist even when it does not. Perceived conflicts of interest can be just as important as actual conflicts of interest in undermining trust in research and researchers.^9^, ^22^, ^23^ This perception is not always unwarranted, with many examples of the food industry donating funds to, or collaborating with, universities as part of a deliberate strategy to improve their reputation and to influence the evidence base.^7^, ^24^, ^25^, ^26^, ^27^

Secondly, the type of interaction that population health researchers may have with the food industry is varied and may have different consequences. Financial transactions, for example, research grants, are the most publicized form of interaction.^7^ These are often supported by government and research institutions, particularly in the face of declining public funds for research.^17^, ^28^ However, interactions may take other forms, such as in‐kind funding, which includes access to resources, travel costs, or honoraria or direct dialogue, which could include providing advice on industry initiatives.^13^ There is limited acknowledgement of the existence of these other forms of interaction or guiding principles about them.

Finally, the “food industry” includes a heterogeneous range of companies and products. Different food products have different consequences for health, and many companies have a mixed portfolio of products. Furthermore, companies themselves may engage in health promoting, health damaging, or health neutral behaviours beyond the products they sell. This heterogeneity makes it difficult to come up with overarching principles for appropriate interactions. To help clarify what constitutes appropriate interactions between population health researchers and the food industry, we aimed to systematically harvest from the literature, synthesize and analyze the key principles that have been identified to help prevent, or manage actual or perceived conflict of interest.

## 2 METHODS

Our protocol was informed by the Arksey and O'Malley framework for conducting a scoping review.^29^ A scoping review is a rapid form of knowledge synthesis designed to identify gaps in the evidence base. It is an iterative process guided by a requirement to identify all relevant literature regardless of study design.^29^ The review protocol was registered with PROSPERO, registration no. CRD42017060539.

### 2.1 Search strategy

We conducted a number of different searches between March and June 2017 to identify relevant peer‐reviewed and grey literature. Firstly, a comprehensive search was conducted on the full holdings of PubMed and SCOPUS databases with no restriction on date or country of publication, although only documents in English were reviewed. The search strategy and inclusion and exclusion criteria can be found in Box 1.

Box 1 **Search strategy, inclusion, and exclusion criteria**

**Search strategy:**

The search strategy included subject heading and text word terms appropriate to each database. Combinations and synonyms of the following terms were used: “conflict* of interest,” OR “public*private partnership*” OR “university‐industry relations” AND “diet” OR “nutrition” OR “food” OR “obesity” OR “food industry” AND “manag*” OR “guiding principle*” OR “codes of conduct” OR “framework” OR “standard.”

**Inclusion criteria:**

Documents that evaluate, compare, use, or describe a process, framework, or practise for identifying and/or preventing and/or managing conflict of interest in private‐public partnerships between population health researchers focussed on diet and/or nutrition and commercial food companies.

**Exclusion criteria:**

Articles, papers, and reports that only describe the concept of conflict of interest or only analyze and report on the existence of a conflict of interest in a private‐public partnership involving population health researchers and a commercial food company.

Articles, papers, and reports that focus on conflict of interest solely in food technology, clinical nutrition, agriculture, food safety, or nutrition policy making with no reference to research.

Articles, papers, or reports in a language other than English.

A Google advanced search using the same search terms, inclusions, and exclusions as above, was undertaken to identify grey literature. The addition of “filetype: PDF” was used to further refine the search as most relevant documents were in a PDF format. Where more than 50 results were found, only the first 50 were scanned, as initial searches revealed no new documents after this.

Additionally, over 200 key informants in the field, identified via international public health nutrition associations and other databases, were contacted and asked to nominate documents for inclusion. Finally, reference lists of included documents were hand searched for additional eligible documents.

**Figure 1:**
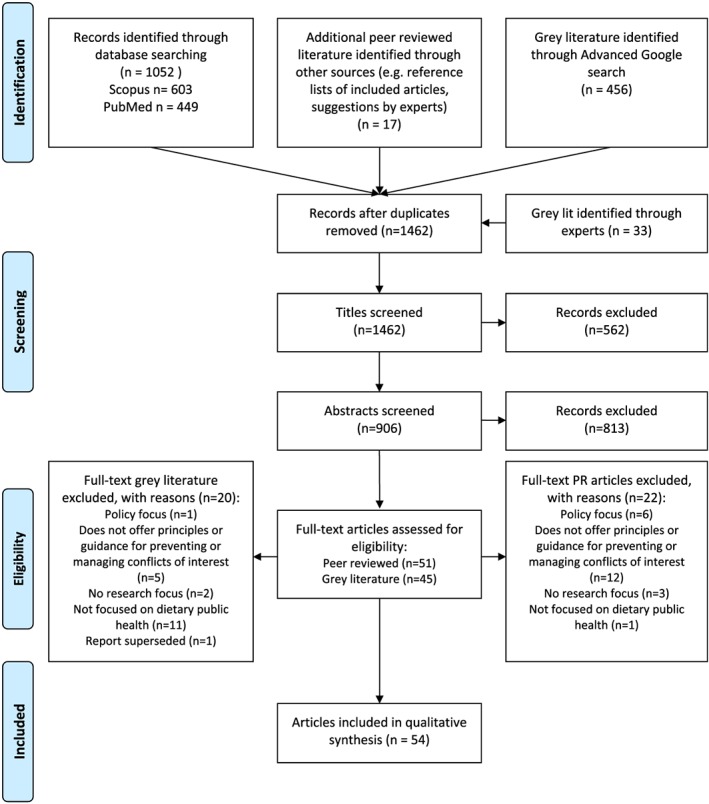
PRISMA 2009 flow diagram [Colour figure can be viewed at wileyonlinelibrary.com]

Database searches were managed using EndNote X5. One author (K.C.) screened retrieved titles and abstracts for eligibility. The full texts of potentially eligible documents were then retrieved and K.C. reviewed these to determine whether they met the inclusion criteria. A second reviewer (M.W.) double screened 15% of included documents. Reasons for excluding documents were recorded, and 15% of these decisions were checked by M.W. Any identified discrepancies were discussed and resolved by K.C. and M.W. A PRISMA flow chart (Figure 1) documents the search and selection process.

### 2.2 Data extraction and synthesis

Documents from all sources were analyzed until concept saturation was reached; that is, no new information, themes, or descriptions of principles for addressing conflicts of interest were found.^30^ To support this, a data extraction form was developed and reviewed by all authors. The extracted data included publication year, country, whether there was a definition of conflict of interest, the principles or activities for preventing or managing conflict of interest, the target users, whether competing interests were disclosed, and whether the reviewer perceived the authors had a conflict of interest based on their place of employment. Data extraction was undertaken by K.C. No formal quality assessment was undertaken following standard practise for scoping reviews.^29^

Key extracted principles were imported into NVivo v11 for analysis.^31^ An inductive process of coding and analysis was used. This allowed the initial codes and then the subsequent themes and names for the themes to flow from the data.^32^ This process was initially undertaken by K.C.; M.W. duplicate coded 15% of included documents. A flow diagram to illustrate how the themes interact was also constructed to aid analysis.

In order to explore the origins and evolution of ideas in this body of literature, we also undertook a network analysis of all citations. NodeXL^33^ was used to visualize the number of times included documents were cited by other included documents.

## 3 RESULTS

We identified 54 relevant documents describing principles for preventing or managing conflict of interest between the food industry and dietary public health researchers. Just over half of these documents were published in peer‐reviewed journals (n = 28; 52%). Documents included were published between 1999 and 2017 with the largest number focused on high income countries (n = 24; 44%). Six (11%) were focused on issues specific to low and/or middle income countries, and the remainder did not specify the audience or context. Forty one percent of documents were clearly in favour of necessary interactions with private industry (n = 22), 24% were against (n = 13), and the remainder took a neutral position. For those documents clearly in favour of private industry interactions, half (n = 11) had either declared funding by the food or beverage industry or had authors who were employed by the food and beverage industry. The most comprehensive guidance for those working in population health was designed for non‐governmental organizations^34^, ^35^ or government agencies considering private investment in nutrition intervention programmes.^13^, ^36^ The most comprehensive guidance specifically designed for researchers was developed by Rowe et al,^15^, ^37^ and this was referred to as an accepted standard in other documents (particularly those with a pro‐industry engagement stance).^10^, ^16^, ^17^, ^38^, ^39^, ^40^, ^41^

**Figure 2:**
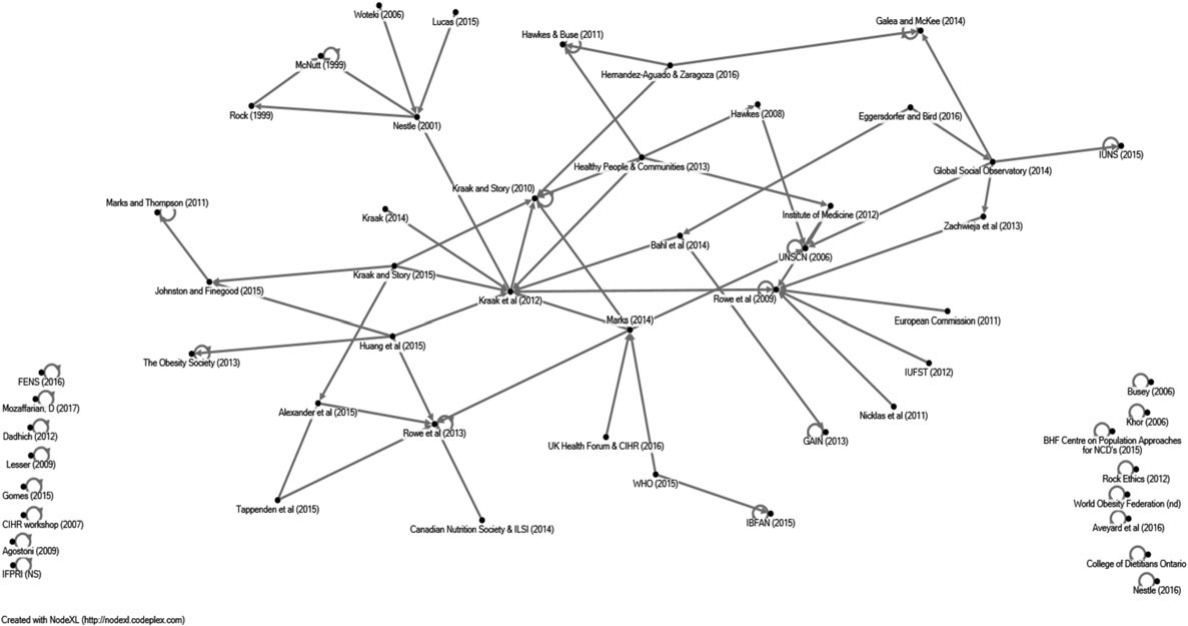
Network analysis of all citations from the systematic scoping review

In the majority of cases, stated principles appeared to be based on authors' opinions, rather than empirical research. Our network analysis of citations (see Figure 2) revealed two documents that had a higher number of citations than the others,^34^, ^37^ although the number of citations were fairly low showing no clear evolution of principles over time. Fifty percent (n = 27) of documents did not cite any other document in the review.

Fifty‐six unique principles for preventing or managing conflicts of interest were identified and synthesized into five themes: (a) structure and governance of funding; (b) undertaking a risk assessment; (c) maintaining high standards of research governance; (d) ensuring high levels of transparency; and (e) improving publication standards (see Table 1).

**Table 1:** Principles for preventing or managing conflicts of interest identified from published sources

Themes, Subthemes, and Statements	Sources
1.Funding	
1.1 A pool of funding from the food industry that is independently administered by a publically accountable third party should be created	13, 42, 43
1.2 A system where industry provides funding to research institutions, not individual researchers or research units, should be created	10, 42, 44
1.3 Researchers should not accept funds from the food industry	7, 14, 45, 46, 47
1.4 Researchers should not accept funds from processed food companies	7, 14, 42, 45, 46, 47, 48, 49, 50
1.5 Researchers should not accept funds from any commercial organization	45, 46
For those who accept funding from the food industry	
1.6 Researchers should have no commercial interest in the product being researched	42
1.7 Funding from industry should reflect the full cost of the research (eg, using a standard academic costing framework) and not more than this amount	42
1.8 Industry funding should be nondesignated	51
1.9 There should be no involvement of the food industry funder in any aspect of a research project	27
1.10 There should be limited involvement of the funder in any aspect of the project	52
2. Undertake thorough risk assessment	
Risk assessment of potential partner(s)	
2.1 Have a clearly identified system to identify and assess interests of potential partners	12, 13, 14, 17, 34, 35, 36, 43, 46, 49, 50, 51, 53, 54, 55, 56, 57
2.2 A partnership should only be initiated if it will help advance the public health goal	4, 14, 15, 17, 54, 58
2.3 Only enlist partners who are committed to long term funding and engagement	15, 16 Opposite sentiment: 35
2.4 Only enlist partners who are committed to sharing of research data arising from the research project	15, 16
2.5 Only enlist partners who operate in an ethical manner and uphold the human rights of women, men, and children	14, 34, 35, 48, 51, 53, 59
2.6 Ensure the organizational values and overarching goals of the partners are compatible	4, 14, 17, 34, 35, 36, 54, 60, 61, 62
2.7 Ensure all partners have shared objectives and a shared approach to the research question and activities	15, 58, 60, 63
2.8 Avoid companies whose objectives and/or goals are related to the increased production, supply or demand of “unhealthy food” products and/or to the promotion of unhealthy and unsustainable ways of eating and producing food	35, 42, 48, 49
2.9 Assess whether the partnership could undermine the integrity or trustworthiness of my institution	17, 35, 36, 54, 55, 62
Risk assessment of type of engagement	
2.10 Consider whether the proposed engagement would be acceptable across institutions and national borders	4, 61
2.11 Be guided by generic international protocols and frameworks (eg, World Health Organization) on appropriate types of engagement	14, 51
Ensure public benefit is at centre of agreement	
2.12 Consider whether the partnership provides maximum benefit to society	12, 16, 36, 50, 54
2.13 Consider what the public would think about this arrangement	17, 27, 46, 59, 61, 62, 64, 65
Consider possibility of reputational damage and loss of trust	
2.14 Consider what my colleagues would think about this arrangement	64
2.15 Decline to give industry sponsored presentations	34
2.16 Do not “ghost write” publications for the private sector	34, 64
2.17 Do not accept gifts or hospitality if it compromises or appears to compromise objectivity	64, 65
2.18 Do not participate in undisclosed paid authorship arrangements in industry‐sponsored publications or presentations	39
2.19 Do not allow the commercial partner to co‐brand (eg, use their logo) on the research project or related material	4, 43, 51
3. Research governance	
3.1 Clearly state and agree goals, objectives, roles, and responsibilities and accountability before work commences	4, 10, 12, 13, 14, 15, 16, 17, 27, 34, 38, 43, 54, 58, 59, 60, 66
3.2 Plan research so it is designed objectively and is scientifically sound in its approach	37, 39, 41, 44, 67
3.3 Establish upfront control and ownership of the data by the researcher/s but provide accessibility to data and analyses to the industry funder	37, 40, 67, 68
3.4 Data analysis should be done by statisticians independent of the researcher/s who designed and conducted the study	8, 42
3.5 Undertake random audits of data provided by food companies for research projects	10
3.6 Secure oversight of the research by a nonconflicted third party	10, 12, 13, 16, 34, 41, 60
3.7 Require all trials or other studies in dietary public health to be registered at time of initiation of the study	8, 45
Ensure partners have equal power	
3.8 Along with the private sector, include members of civil society (eg, foundations, NGOs, and consumers) as partners	10, 15, 16, 17, 54, 60
3.9 Ensure diversity of partners to avoid undue influence of any one partner	12, 13, 14, 15, 16, 17, 43, 51, 52, 69
3.10 The research institution must be able to independently criticize a commercial‐sector entity for issues unrelated to the partnership	34
Ensure public benefit is at centre of agreement	
3.11 Engage independent members of the public in the process of defining research problems and subjecting research projects to ongoing critical scrutiny	15, 39, 43,
Management of conflict(s) of interest	
3.12 Have a clearly identified system to identify, assess, and manage the interests of all stakeholders	12, 13, 14, 34, 36, 43, 51, 54, 55, 56, 59
3.13 Recuse stakeholders from committee (or similar) decision making where there may be an actual or perceived conflict	13, 16, 34, 59, 68, 70
3.14 Continuously monitor for conflicts of interest	13, 17, 39, 43, 51, 55
Consequences	
3.15 Establish clearly stated exit mechanisms for partners	16, 17, 34, 36, 43, 54
3.16 Establish sanctions with effective enforcement for violation of conflict of interest including reprimands, fines, and dismissal	13, 43, 45
4. Transparency	
4.1 Explicitly report funding, governance structures, research frameworks, and findings and ensure it is publically available	10, 13, 14, 27, 69, 71
4.2 All individuals involved in a research partnership should undertake full disclosure including financial, personal, and professional interests over the past 5 years	7, 13, 14, 16, 24, 34, 37, 39, 45, 51, 52, 53, 56, 59, 64, 65, 67, 68, 70, 71, 72, 73 5 years specifically: 8, 69
4.3 All individuals involved in research partnership should disclose interests of their spouse/partner, minor children, employer, and business partners	13, 64
4.4 Ensure all presentations and media releases from an industry partner, regarding any research project to which they have contributed direct or in‐kind funding, are endorsed by the research partner	64, 67, 69
4.5 Require full disclosure of funding sources and financial interests in research media releases	67, 74
4.6 Require a declaration of interests slide in all presentations and a written statement on any poster presentations	39, 40, 52, 56, 67, 70, 74
4.7 Establish a public database of conflicts of interests in dietary public health research	8
5. Publication	
5.1 Academic researchers should include all potential conflict of interests including full affiliation as well as disclosure of industry funding and/or industry affiliation in research publications	37, 39, 40, 67, 68, 70, 73
5.2 Ensure research partner retains full rights to publish all results, including those unfavourable to the funder	27, 37, 40, 42, 52, 67, 68, 69
5.3 Ensure the research partner has control over the preparation and approval of peer‐reviewed manuscript	67, 68
5.4 Establish clear definitions around sponsorships and author affiliations to be used in publications, such as: industry funded, non–industry funded, and unknown/unclear sponsorship	75

### 3.1 Structure and governance of funding

The research funding process frequently featured as a critical component for preventing conflicts of interest. This included two elements: the appropriateness of accepting funds from the food industry and the governance processes for accepting funds. There was a variety of principles related to the appropriateness of accepting funding; these ranged from not accepting any commercial funding^45^, ^46^ to not accepting funding from the food industry^7^, ^14^, ^45^, ^47^ or the ultraprocessed food industry.^42^, ^48^, ^49^, ^50^ Alternatively, for those who believed it was appropriate to accept funding, some suggested that it should go through an independent, intermediary body,^13^, ^42^, ^43^, ^44^ whilst others actively encouraged direct relationships between researchers and the food industry.^10^, ^16^, ^17^, ^27^, ^38^, ^39^, ^40^, ^41^, ^53^, ^60^

For those accepting funding from the food industry, there was also a range of views on the role that the food industry should take in a research project. Several of the statements were ambiguous and provided little detail on the practicalities of this arrangement. For example, there should be “little involvement from the funder”^22^ but with no explanation of what constitutes “little involvement.”

### 3.2 Undertake a risk assessment

For those organizations or researchers considering interacting with the food industry, a key theme was the need to undertake a thorough risk assessment before proceeding. This included not only assessing the suitability of a food company, as well as the type of interaction involved.

In order to assess the suitability of a food company for an interaction, some authors recommended ensuring that a food company had compatible goals or values,^4^, ^14^, ^17^, ^34^, ^36^, ^54^, ^60^, ^61^, ^62^ although limited guidance was provided on how to determine this. Kraak and Story^63^ and Rowe et al^15^ encouraged a more moderate approach for assessing food companies, proposing interactions only with those who share objectives or have a shared approach to the research. Others provided more explicit guidance for assessing potential food companies with which to interact, stating that an interaction should only be initiated if it helps to advance public health goals^4^, ^14^, ^15^, ^17^, ^36^, ^54^, ^58^ and that companies selling or promoting unhealthy foods should be avoided.^35^, ^42^, ^48^, ^49^

In terms of assessing the type of interaction, recommendations considered whether the interaction would be acceptable across institutions and national borders.^4^, ^61^ Generic international protocols and frameworks, such as guidance from the World Health Organization, were also mentioned.^14^, ^51^

Underpinning proposed risk assessments was the possibility of reputational damage and loss of trust. This subtheme included a number of different principles ranging from not ghost‐writing publications for the private sector^34^, ^64^ to not allowing a commercial partner to co‐brand research projects or related material.^4^, ^43^, ^51^

A final subtheme for assessing risk was to ensure public benefit was at the centre of the agreement; this included considering public sentiment regarding the arrangement^17^, ^27^, ^46^, ^59^, ^61^, ^62^, ^64^, ^65^ and whether the partnership maximized benefit to society.^12^, ^16^, ^36^, ^50^, ^51^, ^54^

### 3.3 Maintain high standards of research governance

The majority of documents stated it was essential to plan research objectively and develop a clear research protocol outlining all roles, responsibilities, goals and objectives, and research methods before the research commenced. Several documents encouraged an extra level of accountability through the involvement of independent third parties, either for data analysis or oversight.^8^, ^10^, ^12^, ^13^, ^16^, ^34^, ^41^, ^42^, ^60^ Many of the research governance principles can be considered generic principles of good research practise and not specific to minimizing conflicts of interest.

Another important mechanism of research governance that authors proposed was to ensure that no partner has disproportionate power or influence. Proposed strategies for achieving this included involving members of civil society as partners^15^, ^16^, ^17^, ^36^, ^54^, ^60^, ^76^ and/or ensuring a diversity of partners.^12^, ^13^, ^14^, ^15^, ^16^, ^17^, ^22^, ^43^, ^51^, ^69^

The importance of having a clear system to identify and manage conflicts of interest was highlighted by most documents. Suggestions for achieving this included recusing stakeholders with actual or perceived conflicts from decision making,^13^, ^16^, ^34^, ^59^, ^68^, ^70^ continuously monitoring conflicts of interests,^13^, ^17^, ^36^, ^39^, ^43^, ^51^, ^55^ establishing exit mechanisms,^16^, ^17^, ^34^, ^36^, ^43^, ^54^ and establishing sanctions for violation of conflicts of interest policy.^13^, ^43^, ^45^

### 3.4 Ensuring high levels of transparency

The most common, and sometimes only, strategy noted to prevent and manage potential conflict of interests was full disclosure.^24^, ^65^, ^70^ However, the definition of full disclosure varied. Several documents stated that “conflicts of interest should be declared” without defining a “conflict of interest.”^7^, ^24^, ^56^, ^70^, ^74^ Others provided clarification by specifying that conflicts of interest that were financial, personal, or professional should be disclosed^8^, ^68^, ^72^ or that the interests of the spouse or partner, minor children, employer, or business partners of all involved in the research partnership should also be disclosed.^13^, ^64^ The other element of this theme was ensuring that full disclosure occurred with all elements of research dissemination whether in the form of a project report, journal article, conference presentation, press release, or a public talk.^22^, ^39^, ^51^, ^56^, ^67^, ^70^, ^74^

### 3.5 Improving publication standards

The final theme acknowledged the powerful role that journal editors can play in ensuring standards are met. Aspects of transparency and research governance were central to this theme and included strategies to ensure clear definitions for authorship and affiliation^75^ and control over the manuscript by the research partner.^67^, ^68^ Strong levels of support were seen for the general principle of full disclosure in research publications^8^, ^22^, ^24^, ^37^, ^39^, ^45^, ^51^, ^67^, ^68^, ^70^, ^73^, ^75^ and ensuring the right to publish all results, including those unfavourable to the funder or commercial partner.^15^, ^22^, ^27^, ^37^, ^42^, ^51^, ^67^, ^68^, ^69^, ^74^

**Figure 3:**
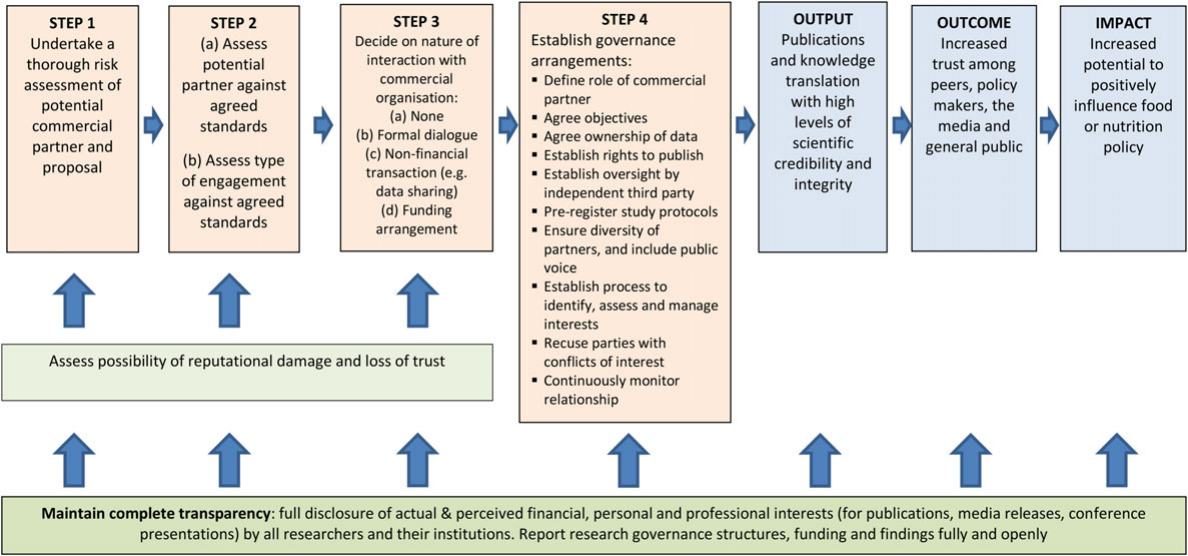
Flow chart identifying steps in process and key principles to help researchers determine whether and how to interact with commercial organizations [Colour figure can be viewed at wileyonlinelibrary.com]

A flow diagram summarizing the researcher decision‐making process was synthesized from the literature, using a process of thematic analysis and theorizing, to aid further analysis (see Figure 3). It demonstrated that there is general agreement on the decision‐making process, but the specifics of decision making for certain steps were unclear.

## 4 DISCUSSION

### 4.1 Principal findings

Our systematic scoping review identified 56 principles from 54 documents. These covered five key areas: governance of funding, risk assessment, maintaining standards of governance, ensuring transparency, and improving publication standards. The review has highlighted the wide range of principles proposed to prevent or manage conflicts of interest between population health researchers and the food industry. A high level of agreement between authors exists on some of these principles, whilst others remain more contentious, particularly those related to whether it is appropriate to interact with the food industry and what kinds of interactions are considered problematic. There was limited guidance on how the principles should be applied in practise.

### 4.2 Relationship to existing knowledge

There was more consensus and detailed guidance related to three themes: “ensuring high levels of transparency,” “maintaining high standards of research governance,” and “improving publication standards.” These encompass established and foundational principles of research and involve unequivocal decision making. So it is perhaps unsurprising that they had high levels of support. However, within the theme “ensuring high levels of transparency,” the concept of “full disclosure” meant different things in different documents. Although there was generally support for individual financial disclosure, acknowledgement of the need for more extensive disclosure was more limited. Agreeing on a standard for full disclosure is important as it provides those reading or listening to findings with a basis for drawing their own conclusions regarding potential for bias and confidence in the findings.^77^, ^78^

The flow diagram (Figure 3) highlights the importance of risk assessment processes in making decisions about interacting with the food industry. However, few documents specified criteria or a structured process to guide this. The lack of specific criteria highlights the moral as well as scientific values that should be considered when assessing these issues.^79^ Without clear guidance in this area, researchers may have trouble conducting risk assessments. Furthermore, the ownership arrangements of many food companies make the assessment of their suitability for research‐related interactions difficult to judge. Does one assess a food product, the wider brand, the company, or its parent company? Clear guidance around this complex issue was not included in any documents. For researchers who are seeking guidance on which commercial organizations to interact with and for what activities, this review has demonstrated that there is neither clear consensus nor guidance on how to assess the risks. More work is needed to clarify these issues.

Few documents recognized the diversity of types of interaction with industry or whether different strategies are required for different types of interaction. Calls for further recognition of the potential heterogeneity of interactions, in particular partnerships, have been made in relation to population health research focussed on diet or nutrition^57^ and other types of research.^50^ However, Johnston and Finegood^4^ acknowledge that as partnerships often require flexibility and exist along a continuum, it may be difficult to establish clear definitions and guidelines for all types. Further exploration is warranted to determine a typology of different interactions and whether different guidance is required for these different types of interaction.

This literature review has highlighted that polarized views on either side of this argument are evident. For authors who clearly supported engagement with industry, there was limited acknowledgement of the risks of this. Most of these documents also failed to acknowledge the potential power imbalance in such relationships or display an understanding of who stands to gain from relationships in the long and short term. Without acknowledging these risks, it is hard to identify and prevent or manage them.^55^, ^57^ Amongst those authors who were clearly against interactions with industry, there was limited acknowledgement of its potential benefits.^42^ Furthermore, there was a lack of acknowledgement that bias can occur from a number of perspectives, and whilst not as extensively documented, this has been found to occur in researchers' opposition to industry interests resulting in overstatement of results.^80^, ^81^ The biases created by this emotive and value‐based environment—both conscious and unconscious—make it difficult for all parties to critically assess the research opportunities offered by interaction with the food industry and related research designs and methods. Finding a way to objectively assess such research opportunities and methods is critical to advancing the integrity and credibility of population health research on diet and nutrition.

### 4.3 Strengths and weakness of the study

Whilst much has been written on the topic of conflicts of interest between researchers and the food industry,^7^, ^26^, ^27^, ^77^ this is the first study to systematically review and synthesize the range of principles that have been proposed to manage relationships between population health researchers and the food industry so as to prevent or minimize conflicts of interest. The comprehensiveness of the search and the use of two independent reviewers for 15% of the screening, exclusions, data extraction, and coding increase the reliability and validity of the results.

It is important to note that the material analyzed in the review was largely narrative in nature and in the form of discussion papers or guidance. It was not clear in most cases whether the principles cited or related guidance were empirically derived. However, the purpose of this review was to identify exhaustively the range of principles that are currently considered in preventing and managing conflict of interest and then to critically analyze and synthesize these. The use of multiple methods to identify eligible documents further increases our confidence that we are likely to have found the majority of relevant documents and principles. It is noteworthy that our network analysis revealed little evolution of principles over time, with low numbers of citations between articles.

It is possible that some documents offering relevant but generic guidance were not included in the review as we excluded documents without a specific focus on population health research on diet or nutrition. The study is also limited by the lack of documents included from low‐ and middle‐income countries, where particular challenges exist concerning lack of research funding and undocumented interactions between researchers and the food industry. Furthermore, some of the documents reviewed were developed for NGOs engaging in research, and we have extrapolated their recommendations to the academic research setting, which may not be appropriate in every case. Finally, we only included documents published in English, and thus some relevant publications may have been missed. Despite these limitations, we are confident that data saturation was reached to adequately describe the principles surrounding this issue.

## 5 CONCLUSIONS AND IMPLICATIONS

In the light of constrained public sector funding for research, particularly in low‐ and middle‐income countries and the proposition that some population health research questions on diet and nutrition would benefit from industry involvement, it is likely that researcher interaction with the food industry will continue and may benefit population health. For those population health researchers considering interaction with the food industry, our findings highlight that there is a level of consensus on the principles relating to standards of research governance, transparency, and publication. However, in relation to assessing the appropriateness of potential industry interaction and the type of interaction, greater clarity is required to ensure that trust in dietary public health research remains high.

One way to achieve greater clarity on these issues is through formal consensus building. In addition, rather than abstract, high‐level principles, clear action‐guiding recommendations for assessing opportunities to interact with the food industry whilst preventing or managing conflicts of interest should be developed for use by researchers, funders, and journals to ensure the credibility and integrity of population health research involving food industry partners.

## AUTHOR CONTRIBUTIONS

M.W., J.A., O.F., and N.F. developed the concept for the research and designed the study. K.C. and M.W. conducted the searches, study selection, and data extraction and drafted and revised the paper. K.C., M.W., J.A., O.F., and N.F. interpreted the results. All authors critically reviewed drafts and approved the final version of the manuscript.

## FUNDING INFORMATION

M.W., J.A., N.F., O.F., and K.C. are funded by core grants to Medical Research Council (MRC) Epidemiology Unit, University of Cambridge and the Centre for Diet and Activity Research (CEDAR). CEDAR is a UKCRC public health research centre of excellence with funding from the British Heart Foundation, Cancer Research UK, Economic and Social Research Council, MRC, National Institute of Health Research and the Wellcome Trust. NGF acknowledges MRC funding (MC_UU_12015/5). The views expressed in this paper do not necessarily represent those of any of the above named funders.

## CONFLICTS OF INTEREST

The authors have no financial relationships with any organizations that might have an interest in the submitted work. To accord with the principles espoused in this research, we have prepared a detailed declaration of interests for all co‐authors, which can be found in a supplementary file.

## Supporting information

Data S1 Supporting information
